# Single molecule tracking reveals spatio-temporal dynamics of bacterial DNA repair centres

**DOI:** 10.1038/s41598-018-34572-8

**Published:** 2018-11-06

**Authors:** Thomas C. Rösch, Stephan Altenburger, Luis Oviedo-Bocanegra, Miriam Pediaditakis, Nina El Najjar, Georg Fritz, Peter L. Graumann

**Affiliations:** 1 SYNMIKRO, LOEWE Center for Synthetic Microbiology, Marburg, Germany; 2 Department of Chemistry, Philipps Universität Marburg, Marburg, Germany; 3 Department of Physics, Philipps Universität Marburg, Marburg, Germany

## Abstract

Single-particle (molecule) tracking (SPT/SMT) is a powerful method to study dynamic processes in living bacterial cells at high spatial and temporal resolution. We have performed single-molecule imaging of early DNA double-strand break (DSB) repair events during homologous recombination in the model bacterium *Bacillus subtilis*. Our findings reveal that DNA repair centres arise at all sites on the chromosome and that RecN, RecO and RecJ perform fast, enzyme-like functions during detection and procession of DNA double strand breaks, respectively. Interestingly, RecN changes its diffusion behavior upon induction of DNA damage, from a largely diffusive to a DNA-scanning mode, which increases efficiency of finding all sites of DNA breaks within a frame of few seconds. RecJ continues being bound to replication forks, but also assembles at many sites on the nucleoid upon DNA damage induction. RecO shows a similar change in its mobility as RecN, and also remains bound to sites of damage for few hundred milliseconds. Like RecN, it enters the nucleoid in damaged cells. Our data show that presynaptic preparation of DSBs including loading of RecA onto ssDNA is highly rapid and dynamic, and occurs throughout the chromosome, and not only at replication forks or only at distinct sites where many breaks are processes in analogy to eukaryotic DNA repair centres.

## Introduction

SPT/SMT has revealed unprecedented insights into the mechanism of diverse cellular processes such as signal transduction^1^, chromosome segregation^2,3^, transcription^4^, translation^5^, replication^6^ and DNA-repair^7–9^. In bacterial cell biology, SPT data were usually generated by following the fate of multiprotein complexes using time-lapse microscopy and conventional wide field illumination. With the advent of single-molecule localization microscopy (SMLM), there is an increasing number of studies reporting the dynamics of single molecules at the millisecond range and at high optical resolution^10,11^. Generally, dynamics of single-particles are analyzed regarding their type of motion and their diffusive state (directed, sub- or super-diffusive, Brownian and confined), their binding kinetics and their spatial distribution in the cell. Hence, in contrast to ensemble measurements such as fluorescence correlation spectroscopy (FCS) and fluorescence recovery after photobleaching (FRAP) experiments, SMT provides details about individual molecules and their diffusive behavior^12^.

**Figure 1:**
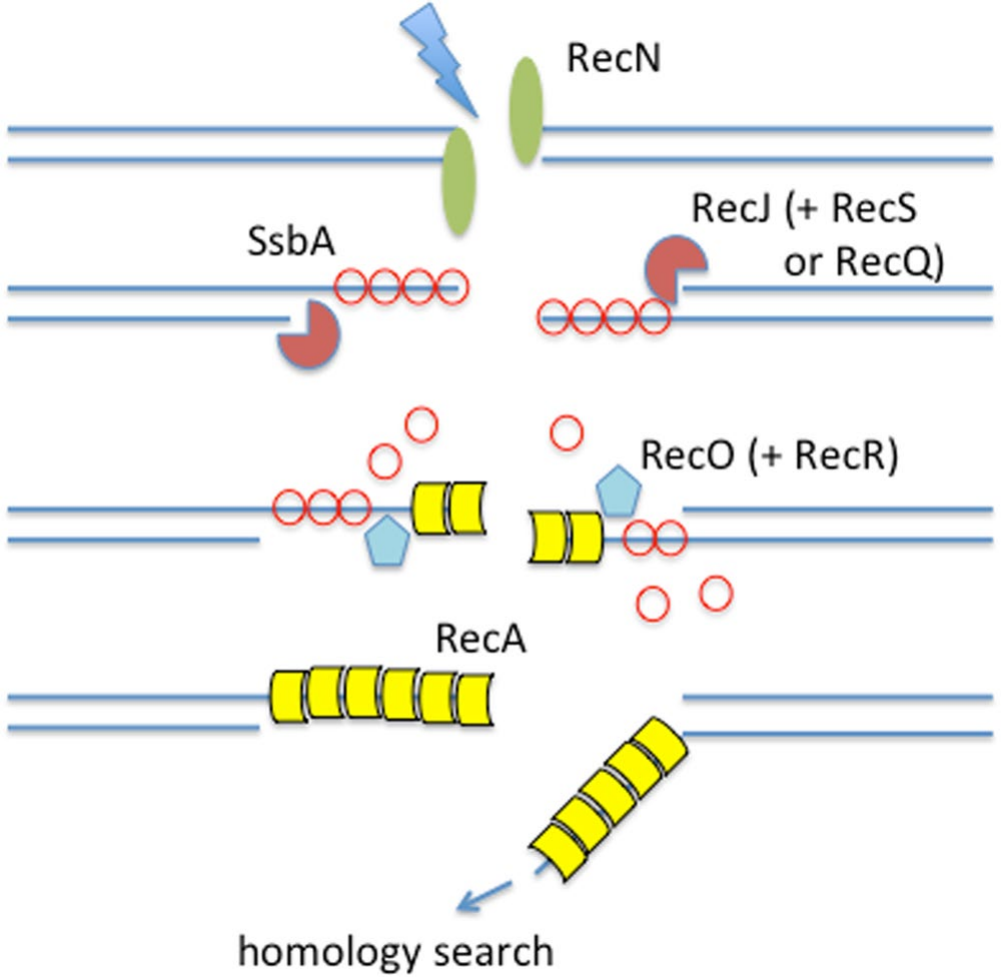
Model for early steps during the repair of double strand breaks (DSBs) via homologous recombination (“presynapsis”). RecN visibly assembles at sites of DSBs (indicated by the flash) at a very early time point, and binds to 3′ ends. RecJ endonuclease (or alternatively the AddAB complex) resects one strand, helped by helicase RecQ or RecS. ssDNA is bound single strand-binding protein SsbA (SSB in *E*. *coli*), which needs to be displaced by RecO and RecR to load RecA onto ssDNA. The RecA nucleoprotein filament is now ready to search for a homologous DNA duplex on the sister chromosome for the formation of a crossover.

Here we have performed single-molecule imaging of proteins involved in early DNA repair reactions at double-strand breaks (DSBs) during homologous recombination (HR), the main mechanism responsible for the maintenance of genome integrity during vegetative growth. DSBs arise either endogenously, e.g. at the replication fork, or exogenously through UV irradiation or genotoxic agents^13^. While unrepaired DSBs lead to the death of all living cells, mis-repaired DSBs might result in malignant transformation of cells. The mechanistic process of HR is conserved in all kingdoms of life and principally requires (a) loading of the recombinase (RecA in bacteria) onto DNA, (b) homology search and strand pairing by the recombinase and (c) resolution of the paired DNA sequences^14^. Process (a) is called pre-synapsis and involves 5′ to 3′ exonucleolytic degradation of DNA ends yielding long 3′-single-stranded DNA overhangs (catalyzed e.g. by RecBCD/AddAB/RecJ enzymes), which are required for loading of recombinase RecA (facilitated by RecO and RecR) that finally pairs the 3′-ssDNA overhang with the non-broken homologous DNA (e.g. RecFOR^15^) (Fig. 1). In bacteria, the initial protein to visually assemble on the nucleoid in response to the induction of DSBs is the structural maintenance of chromosomes (SMC)-like protein RecN^16,17^, which binds to DNA ends *in vitro*^18^ (Fig. 1). No matter what dosages of DNA-damaging agents are added, RecN forms a single assembly on the chromosomes, to which later, RecO, RecF and RecA are recruited^16,19^. Based on these findings, it was suggested that RecN forms repair centers, where multiple DNA breaks can be repaired^16^. However, to date it is not clear how RecN finds DNA breaks, and how it cooperates with downstream events of DSB processing and RecA loading. Therefore, we visualized single molecule dynamics of functional^16^ fluorescent protein fusions of RecN, RecJ and RecO in *Bacillus subtilis* cells before and after induction of DNA damage, using DNA-damaging agent Mitomycin C (MMC). We thereby obtained detailed and quantitative information on changes in diffusion patterns that are different from all proteins investigated, revealing that RecN and RecO change their diffusive behavior, massively entering the chromosome after induction of breaks, thereby maintaining a considerable pool of molecules that continuously scan the DNA for arising breaks throughout the entire genome. This mode of action ensures surveillance and safeguarding of all genetic information.

## Results

RecN and RecO form visible subcellular assemblies on the chromosome 15 to 30 minutes after induction of DNA double strand breaks (DSBs). A single assembly can be seen in a maximum of 70% of cells, no matter how many breaks arose due to added chemicals^17^. Major questions in this process are how RecN and RecO move within cells, at how many sites they assemble, and how long-lived subcellular assemblies are. To study protein dynamics at short time scales, SMT has arisen as most powerful technology. Two main setups are currently used, either stochastic photoactivation of single or few fluorescent proteins (FPs), or bleaching of all FPs until only few or one active FP remains that can be tracked at high speed.

**Figure 2:**
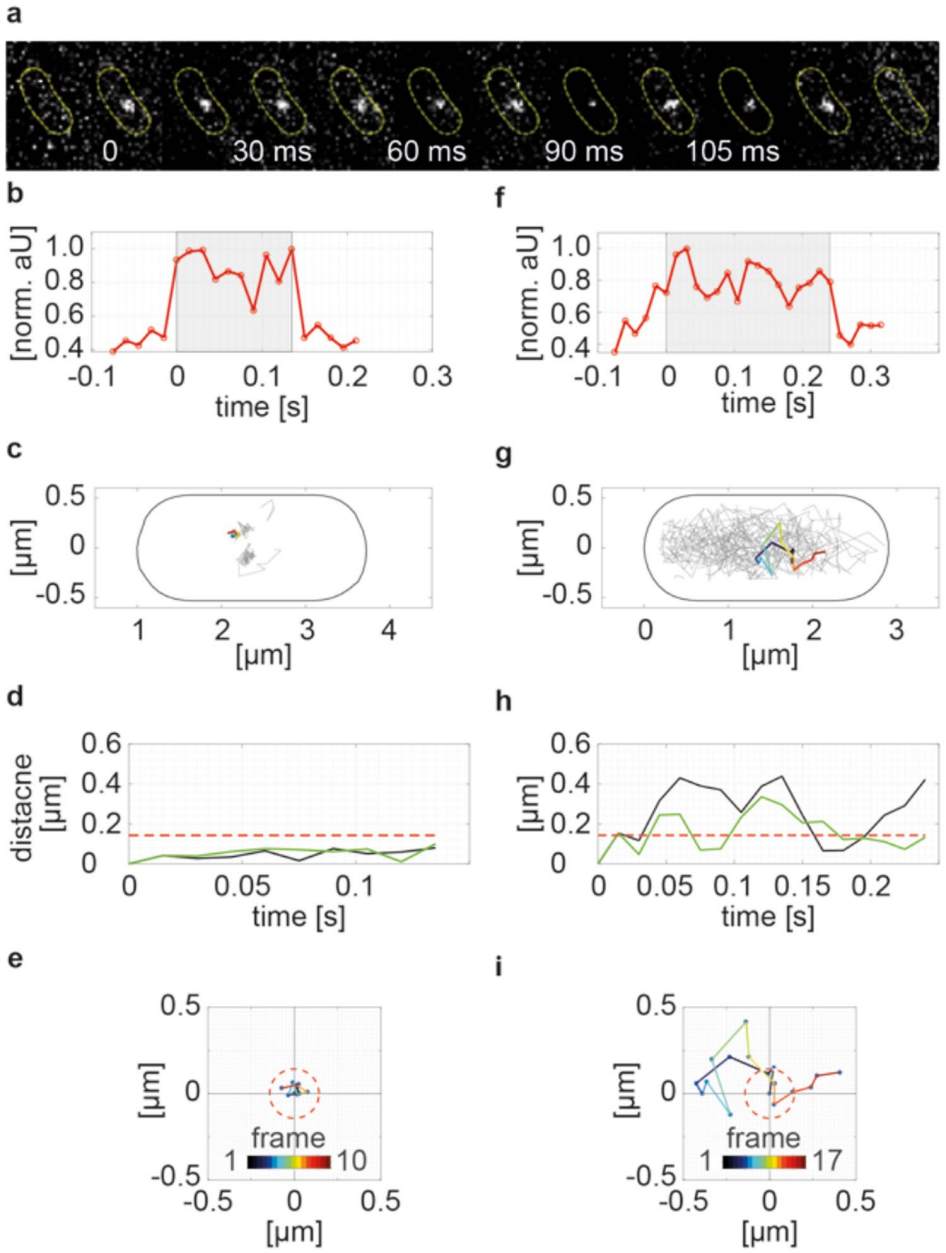
Single-molecule tracking (SMT) of RecN-YFP. (**a**) Example of a single RecN-YFP molecule acquired with an integration time of 15 ms. (**b**,**f**) Normalized signal intensity at the site of localization. The signal was measured starting 5 frames before the molecule appears and ends 5 frames after the molecule disappears in a single step. The grey shaded area highlights the time of appearance of the molecule. The intensity drawn in (**b**) corresponds to the trajectory shown in the montage in (**a**). (**c**,**g**) Projection of all recorded tracks into the coordinate system of the cell. The trajectories shown in (**b**,**f**) are highlighted and color-coded according to its length. (**d**,**h**) Displacement of the trajectories over time. Shown is the Euclidian distance of the molecules to the site of appearance (black line) and its sequential displacements between consecutive frames (green line). The dotted red line represents an upper threshold setting the limit for immobile displacements. (**e**) and (**i**) Color-coded displacements of the trajectories over time. The radius of the circle corresponds to the limit for a track to be considered as immobile.

A prerequisite of SMLM and SMT is a low signal-to-noise ratio (SNR) and a low density of single-molecules in order to localize the molecules at nanometer resolution. Similar to previous studies^2,6^, we used continuous slim field illumination^11^ at an excitation wavelength of 514 nm to increase the SNR, and followed an initial bleaching protocol to diminish the density of the fluorophores in the cell to about 1 to 3 fluorophores per frame. This imaging mode allowed us to acquire individual molecules of functional yellow fluorescent protein (YFP) fusions of RecN, RecJ and RecO with an exposure time of 15 ms per frame at a resolution of ~20 nm (Fig. 2a,b). We continued imaging until all FP fusions in the cell were bleached and subsequently localized and linked the positions of single-molecules with the u-track algorithm^20^. We used C-terminal YFP fusions to RecN and to RecO, which have been described to functionally replace wild type proteins, and are expressed from the original gene locus, as sole source of the proteins in the cell^16,21^. To visualize RecJ, we generated a C-terminal YFP fusion, which was integrated into the original gene locus, and which is likewise expressed as sole source of the protein, and is under the control of the original promoter. We treated cells with different concentrations of MMC for 60 minutes and plated cells, to study if the survival rate is affected by the fusion. While cells carrying a deletion of *recJ* are highly sensitive to MMC, RecJ-YFP expressing cells show wild type-like survival (suppl. Fig. S1), showing that the allele also complements for the function of RecJ.

### DNA-bound and diffusing fractions of RecN, RecO and RecJ molecules change markedly after induction of DNA damage

**Figure 3:**
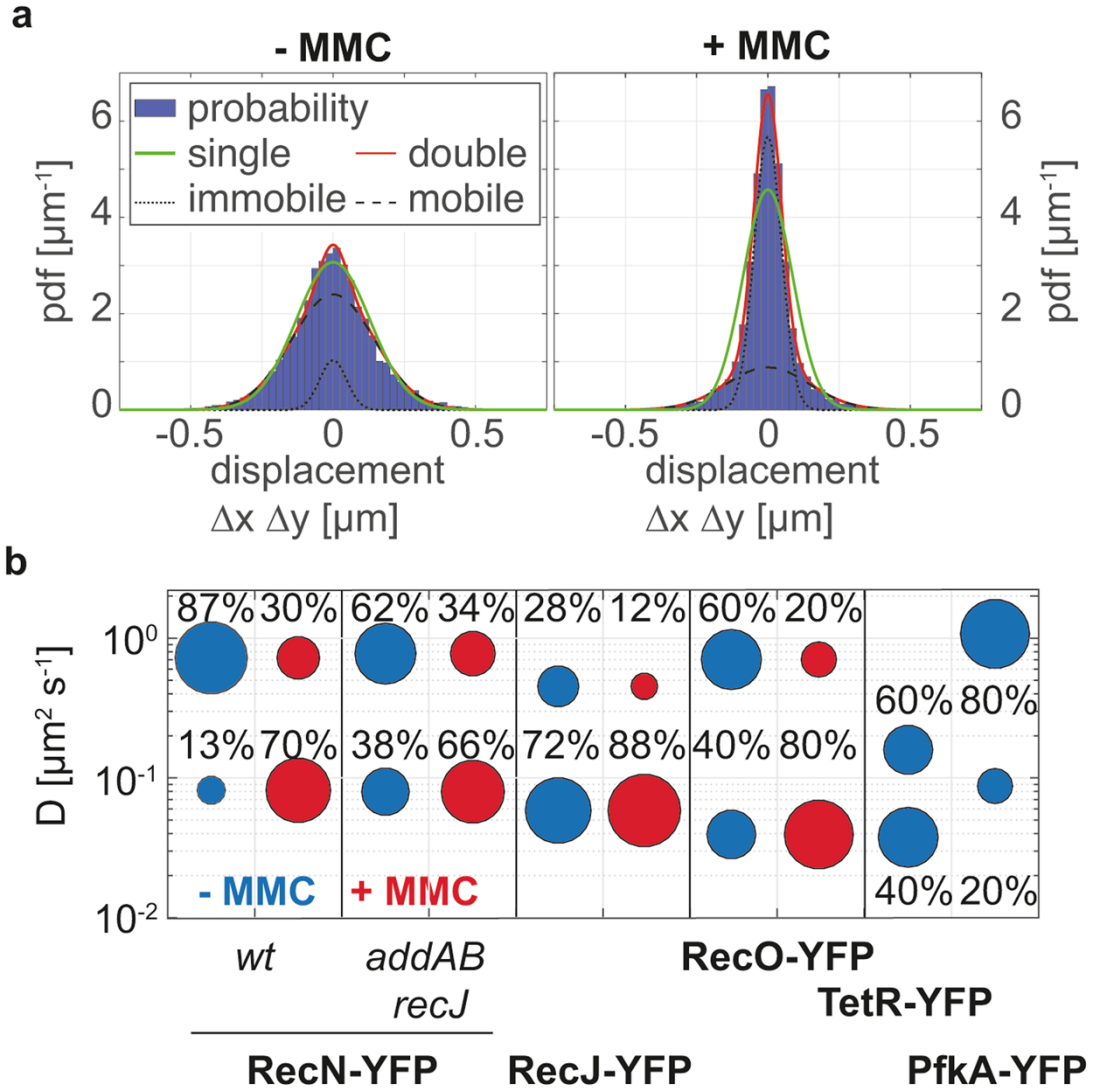
Comparative analysis of diffusive behavior. (**a**) Simultaneous comparison of frame-to-frame displacements for RecN-YFP before and after treatment of the cells with Mitomycin C (MMC). The dotted and dashed lines indicate the distributions of the immobile and mobile fraction. The red line represents the fit deriving from the sum of the two normal distributions and the green line depicts the fit assuming a single normal distribution. (**b**) Bubble plot showing the diffusion coefficients (y-axis) and fraction size (size of the bubble, also stated in percentage above the bubbles) of the fluorescent fusion proteins. Blue bubbles represent untreated samples and red bubbles show samples after treatment with 50 ng/ml MMC. R-square test shows highly significant changes (p < 0.01) between all pairs of +/−MMC.

We employed several strategies to analyze protein dynamics of DNA repair proteins. Firstly, we assessed intracellular protein mobility by applying a Gaussian Mixture Model to the distribution of positional displacements between consecutive time frames (Fig. 3). The model assumes that molecules exist in different diffusive states, e.g. free vs. DNA-bound, and that the fraction of molecules in each state may change between experimental conditions. Simultaneously fitting the width and areas of the Gaussians for both sample conditions yields estimates for the diffusion coefficients, D_1_ (immobile) and D_2_ (mobile) and for the fraction of mobile molecules before (f_2,−MMC_) and after (f_2,+MMC_) MMC treatment. In exponentially growing cells, RecN-YFP showed a high fraction of mobile molecules (f_2,−MMC_ = 87%) moving with D_2_ = 0.72 µm^2^s^−1^ (Fig. 2f–i) (Table 1). After treatment of the cells with MMC, the mobile fraction decreased (f_2,+MMC_ = 30%) and most molecules became immobile with D_1_ = 0.08 µm^2^s^−1^ (Fig. 2b–e and Fig. 3a,b) (Table 1). In accordance with previous epifluorescence microscopy studies, the fraction of immobile RecN-YFP molecules increased early after induction of DNA damage (30 min) and started to saturate at the sublethal dose of 50 ng/ml MMC (Supplementary Fig. S2).

**Table 1:** Results of Gaussian Mixture Model based analysis.

	RecN-YFP		RecN-YFP *addAB recJ*		RecJ-YFP		RecO-YFP		PfkA-YFP	TetR-YFP
MMC	**−**	**+**	**−**	**+**	**−**	**+**	**−**	**+**	**−**	**−**
A1^a^ (%)	13.1	69.7	37.8	66.5	71.9	88.2	39.8	79.3	20.5	59.7
D1 µm^2^/s	0.080		0.0791		0.0591		0.053		0.087	0.051
A2^b^ (%)	86.9	30.3	62.2	33.5	28.1	11.8	60.2	20.7	79.5	40.3
D2 µm^2^/s	0.719		0.7661		0.4485		0.935		1.06	0.21
**τ** [s] (%)*	0.015	0.048	0.027	0.033	0.064	0.086	0.028	0.049	0.015	0.062
**τ**_1_ [s] (%)*	0.010 (77.9)	0.024 (68.3)	0.025 (95.3)	0.021 (78.3)	0.051 (85.1)	0.081 (97)	0.026 (96.3)	0.036 (84.7)	0.010 (82.3)	0.041 (83.2)
**τ**_2_ [s] (%)*	0.038 (22.1)	0.134 (31.7)	0.101 (4.7)	0.116 (21.7)	0.175 (14.9)	0.518 (3)	0.231 (3.7)	0.215 (15.3)	0.055 (17.7)	0.327 (16.2)
C1** (%)	90	30	70	76	47	35	67	53	92	66

*Radius of confinement r = 120 nm, **τ**_1_ = Residence time of static fraction, **τ**_2_ = residence time of static fraction.

**Results of confinement analysis.

^a^Static fraction, % of molecules that have an average diffusion constant of D1.

^b^Mobile fraction, % of molecules that have an average diffusion constant of D2.

To gain insight into the interplay between RecN and end-processing enzymes, we imaged RecN-YFP molecules in the absence of both exonucleases, *addAB and recJ* (Fig. 1). Although the diffusive properties of each fraction were comparable to the wt background (D_1_ = 0.079 µm^2^s^−1^; D_2_ = 0.77 µm^2^s^−1^), the fraction of immobile RecN-YFP molecules was elevated by 25%, even in the absence of MMC (f_1,−MMC_ = 38%; Fig. 3b), while it reached similar level as in the wt background after MMC treatment (f_1,+MMC_ = 67%; Fig. 3b). Therefore, generation of long 3′ DNA ends is not required for DSB recognition by RecN-YFP. Contrarily to RecN, RecJ-YFP did not show a change in its diffusive behavior after MMC treatment, and overall showed smaller displacements no matter if cells were treated with MMC or not (f_1,−MMC_ = 72% vs. f_1,+MMC_ = 88%, D_1_ = 0.0591 µm^2^s^−1^). RecO reacted to DNA damage in a similar way as RecN-YFP by doubling the amount of slowly moving molecules to ~80% in cells treated with MMC (D_1_ = 0.053 µm^2^s^−1^) compared to normally growing cells that show 60% of fast moving molecules with D_2_ = 0.94 µm^2^s^−1^.

To determine whether the range of diffusion constants found for the immobile fractions of RecN, RecJ, and RecO (D_2_ = 0.053–0.08 µm^2^s^−1^) is compatible to a DNA-bound state, we tracked the movement of a chromosome region by using a fluorescent reporter operator system (FROS), in this case repressor protein TetR labelled with YFP and an array of its specific operator binding sites (*tetO*) inserted close to the origin of replication (*oriC*). We report that about 60% of the molecules were bound to DNA with D_1_ = 0.051 µm^2^s^−1^ and 40% moved with D_2_ = 0.21 µm^2^s^−1^ (Fig. 3b) likely scanning along DNA for specific binding sites. To show that our imaging regime also allows tracking of proteins that freely diffuse in the cytoplasm, we imaged the glycolytic protein phosphofructokinase, PfkA. For PfkA-YFP we found molecules moving with D_2_ = 1.1 µm^2^s^−1^ (f_2,−MMC_ = 79%), which is slightly faster than for all other measured proteins in this study. We also detected a small fraction of slow molecules with a D_1_ = 0.087 µm^2^s^−1^ (f_1,−MMC_ = 21%), which seems to bind to an unknown target, or bumps into the membrane (especially at the cell poles).

### Residence time of RecN is increased in cells having DNA damage

**Figure 4:**
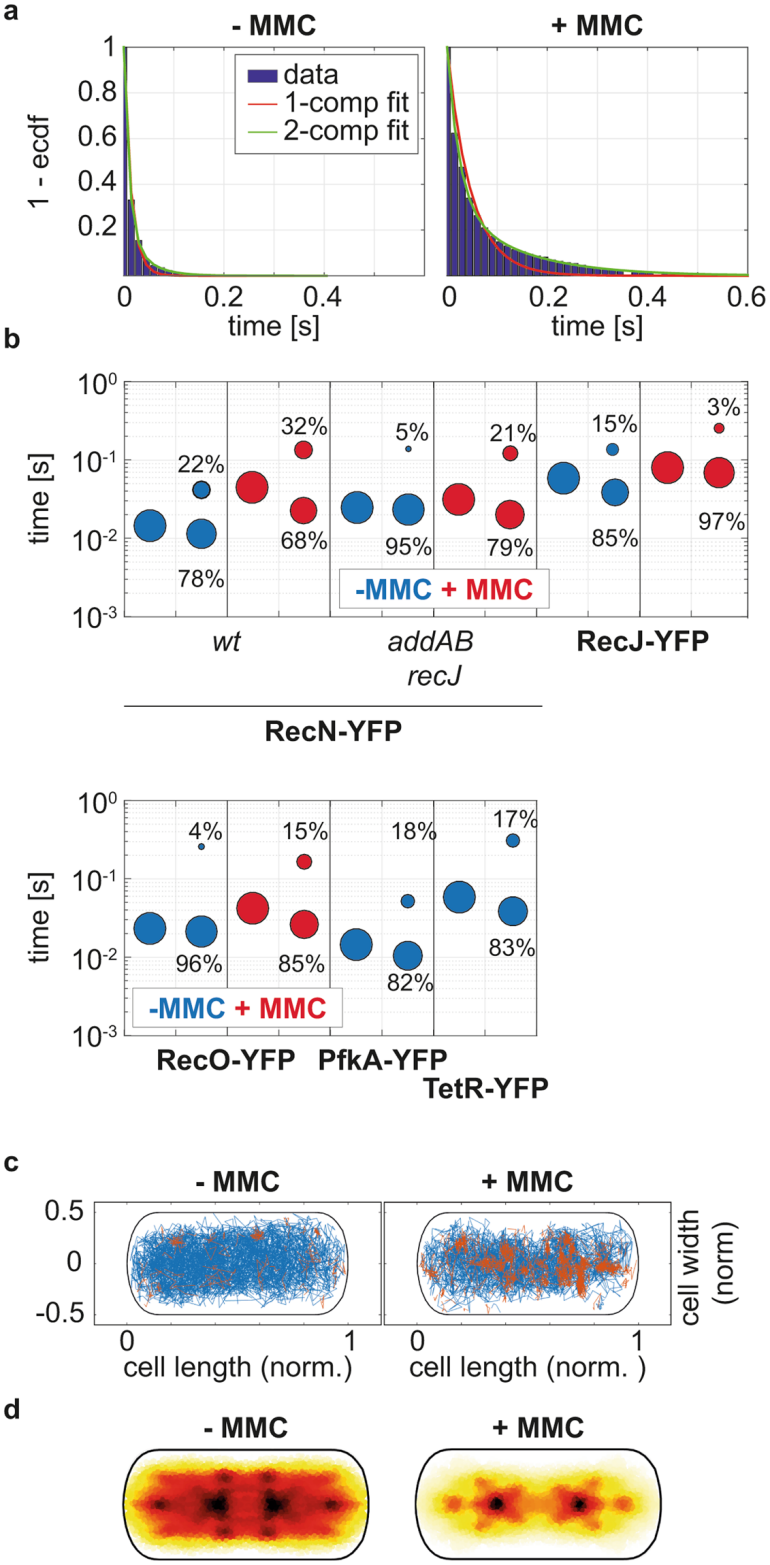
Residence time and spatial distributions of the molecules. (**a**) Cumulative distribution of residence times of RecN-YFP before and after treatment with MMC. Histograms show events of resting fitted either by a one- or two-component exponential function (red and green line). (**b**) Residence lifetime of fluorescent fusions of RecN, RecJ, RecO, PfkA and TetR. Times of resting before treatment with MMC are shown in blue and resting times of molecules after treatment with MMC are shown in red. Results of the 1- (single bubble) and 2 - (two bubbles) component fits are shown side-to-side, the size of fractions for the two populations are stated as percentage above the bubbles. All changes between exponentially growing and MMC-treated cells were found to be highly significant, R-square test (p < 0.01). (**c**) Spatial distribution of RecN-YFP molecules in a standardized cell before and after induction of DNA damage. Mobile molecules are plotted in blue and molecules that remained inside a circle with radius r = 120 nm over their complete lifetime are plotted in red (see track as example in Fig. 1e). (**d**) Heat map of all RecN-YFP molecules before and after induction of DNA damage.

We further calculated the residence times of the DNA repair proteins bound to DNA, by measuring the time a molecule stays within a predefined radius. We defined the radius of the circle based on the immobile fraction of TetR-YFP molecules and set the threshold such that it includes 99% of the smaller displacements (3*σ ≅ 120 nm; see Fig. 2d,e). Corresponding to the increased fraction of immobile molecules, RecN-YFP also showed increased residence times after treating cells with MMC. Before treatment, RecN-YFP could be well fit using a single component decay model giving an average residence time of 0.015 s – corresponding to a single imaging frame (Fig. 4a left panel). After MMC treatment, the cumulative residence time distribution of RecN-YFP could no longer be fitted with a single decay, but instead with a superposition of two exponential decay functions with distinct residence times. Thereby, we estimated that 69% of the residence times were 0.023 s long, while 31% of the binding events lasted for 0.134 s (Fig. 4a right panel). The deletion of the end-processing exonucleases (Fig. 1), *addAB* and *recJ* – leading to the accumulation of DSB even in the absence of DNA damaging agents – increased the residence time of RecN-YFP independent of MMC treatment (Fig. 4b) reinforcing the idea that RecN binds to DSBs in the absence of long 3′ DNA ends. Similarly to RecN, the residence times of RecO-YFP and RecJ-YFP molecules was best described by the superposition of two exponential decay functions. For RecO the fraction of molecules displaying longer residence times increased under MMC treatment. However, for RecJ-YFP the fraction of molecules with longer residence time decreased from 29% to 10% in the presence of DNA damage, albeit this coincides with an increase of the residence time from 0.128 s to 0.256 s (Fig. 4b). These data suggest that RecJ is released from the replication forks, where it is bound by SSB^20^ to act at DSBs occurring on the chromosome away from the replication machinery: this can be seen when all frames of movies are overlaid – revealing the presence of static fractions of RecJ, the number of which increases after addition of MMC (Supplementary Fig. S3). Different from HR proteins, the freely diffusing PfkA-YFP mostly rested in the same radius for only 1 frame (82%). It also showed few, longer resting of ~4 frames (18%), which are likely due to random collisions with the cell membrane (as for any freely diffusing molecules). In contrast, TetR-YFP remained at least ~4 frames at the same site, and rested on average for 0.31 s (~21 frames) at the used acquisition speed, when the distribution was fitted with the two-component exponential decay function (Fig. 4b and Table 1). Of note, actual dwell times of proteins, especially for TetR, are underestimated because of the fast acquisition rates.

### RecN binding events occurs on the periphery of the nucleoid, and RecN enters the nucleoid upon DNA damage induction

**Figure 5:**
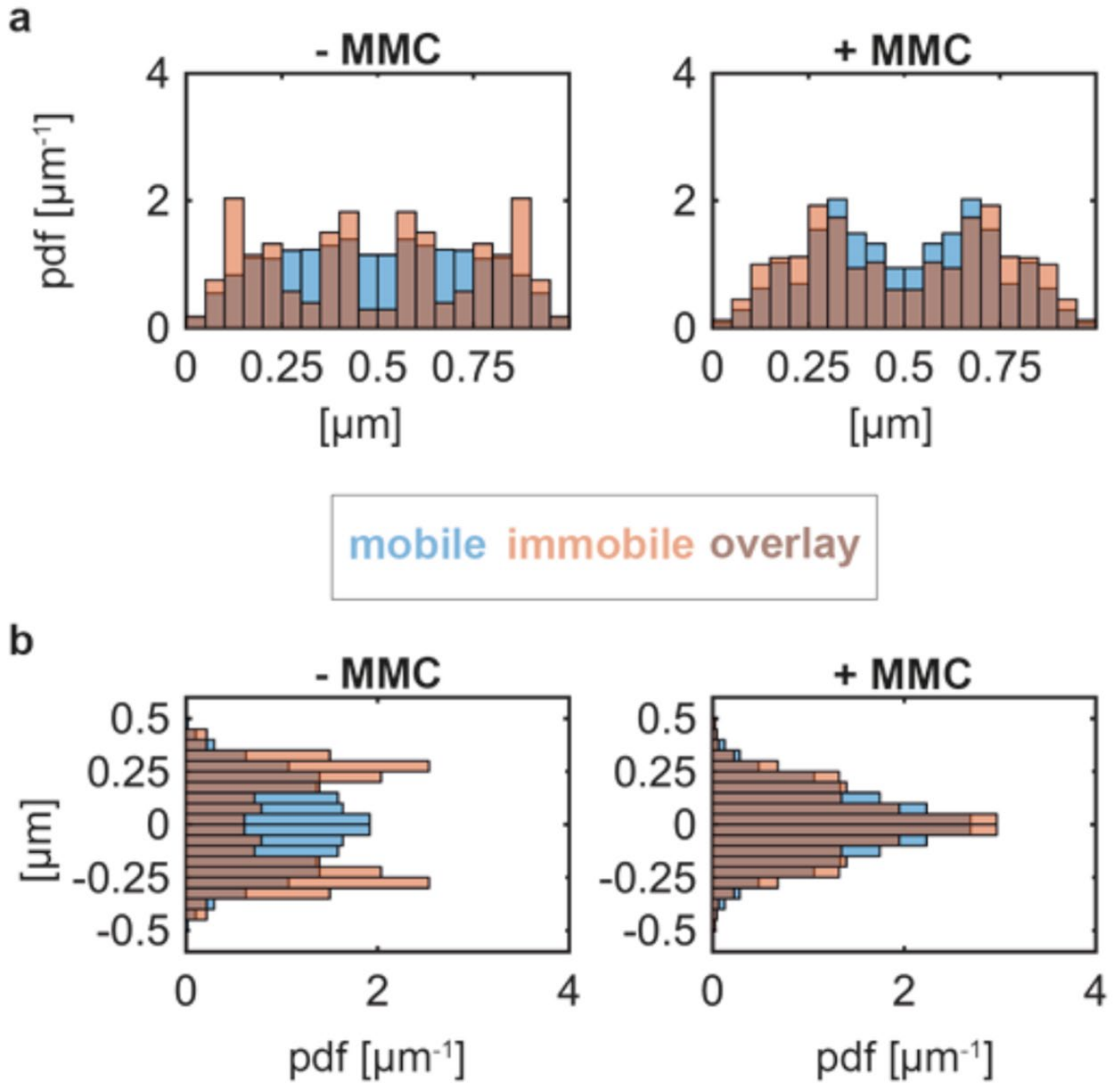
Spatial distribution of single RecN-YFP molecules classified as immobile and mobile. 1D Histograms of tracks binned to the long (**a**) and short axis of the cell (**b**). Left and right panel show distributions in absence and presence of DNA damage, respectively.

**Figure 6:**
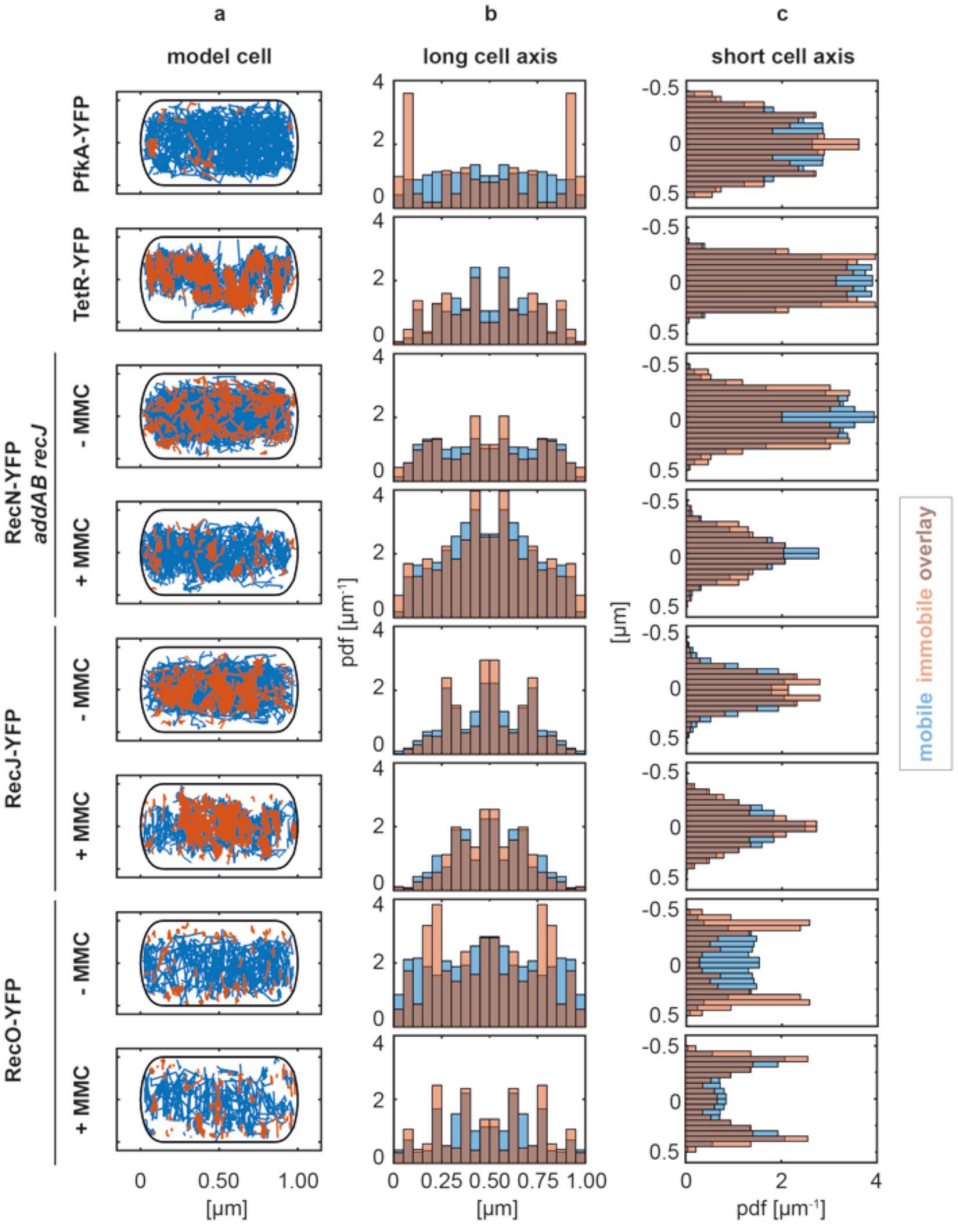
Spatial distribution of classified single molecules trajectories (based on SMT). (**a**) Localization of mobile and immobile trajectories within a model cell with standardized geometry. (**b**) 1D histogram of the classified trajectories binned to the long axis of the cell. (**c**) 1D histogram of the classified trajectories binned to the short axis of the cell. We analyzed >195 trajectories for each condition (see Supplementary Table S3).

We visualized the spatial distribution of mobile and immobile molecules, revealing information about how and where DNA repair proteins search and find DNA target sites. We first classified each trajectory according to the total distance the molecule moves from its primary site of appearance and then binned its localizations into a 2D histogram with a size corresponding to a standardized cell. If the molecule stays inside a circle with radius r = 120 nm over its entire lifetime, the trajectory is classified as immobile (Fig. 2e). If the molecule leaves the circle, the trajectory is considered mobile (Fig. 2i). Note that mobile and immobile tracks from many cells are projected into the standardized cell, while any given cell usually just has mobile or single immobile (i.e. RecN-YFP foci in epifluorescence) molecules after induction of DNA damage. Before treating cells with MMC, mobile RecN-YFP molecules (~90%, blue tracks) were uniformly distributed along the short and long axis of the cell, but with a dip towards the edges of the cell (Fig. 4c, Fig. 5a,b). This is different from freely diffusing molecules of PfkA-YFP (~92%, Fig. 6a), which moved throughout the entire cell, with less of a dip at the poles (Fig. 6b). These data support the idea that a large fraction of RecN molecules moves over the nucleoids, while a smaller one is freely diffusive. Interestingly, immobile RecN-YFP molecules mostly localized to the edges of the nucleoids (Fig. 4c), which can also be seen in the distribution of molecules in x and y directions (Fig. 5a and b). Note that proteins that localize to the cell poles, such as SpoIIIE or SftA, show accumulations very close to the edges of the cell^22^, while there is a clear dip in molecule numbers close to “0” or “1” µm (Fig. 5a) or close to “0.5” and “−0.5” µm (Fig. 5b) for RecN.

Strikingly, RecN molecules relocalized in cells treated with MMC by showing an overall more central localization in the heat map (Fig. 4d), and with many more static tracks present on the nucleoids (Fig. 4c right panel). Figure 5a and b clearly shows that static tracks relocated from the edges of the nucleoids to deep inside the nucleoids. Operator-bound TetR-YFP molecules also localized around the mid-line of the short cell axis and between the mid-cell and quarter positions (Fig. 6b). A peak at the centerline of the short axis was also seen for RecN-YFP in cells deleted for the exonucleases AddAB and RecJ treated with MMC (Fig. 6c), and for RecJ-YFP (Fig. 6d). Along the long cell axis, RecJ-YFP exhibited peaks of mobile and immobile molecules at mid-cell and the quarter positions in normally growing cells, in agreement with its localization at the replisome through its interaction with SsbA^20^. After treatment with MMC, the localization peaks of RecJ-YFP condensed towards the middle of the cell, while PfkA occasionally became static close to the cell membrane (Fig. 6a).

For RecO, induction of DNA damage led to a similar change in localization pattern as for RecN: immobile RecO-YFP molecules accumulated on the nucleoid (Fig. 6e) similar to RecN (Fig. 5b), while during exponential growth, immobile RecO molecules accumulated at the nucleoid edges (Fig. 6b,c). These spatial distribution analyses reveal that RecJ exhibits nucleoid-like localization similar to TetR-YFP, no matter if cells were treated with MMC or not, whereas RecN and RecO significantly changed their pattern of spatial distribution after MMC treatment. Our results reveal that mobile RecN and RecO molecules constantly scan the nucleoid, mostly at its periphery, for DNA damage, but move much deeper into the nucleoid, where they accumulate at any position, upon induction of DNA damage. Such a change in diffusion and in binding pattern has so far been described only for translesion DNA polymerase Pol IV, which accumulates at replication forks but also at other sites on the nucleoids upon induction of DNA damage^23,24^.

### Half-life of RecN assemblies is 2.5 seconds

**Figure 7:**
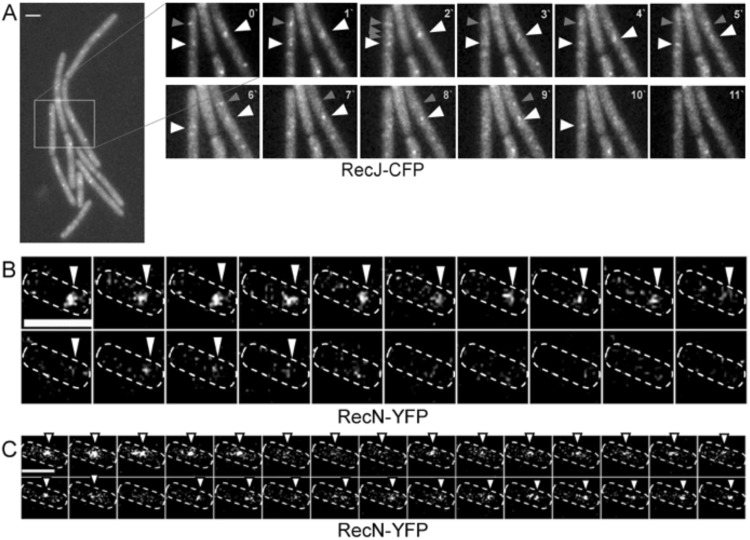
Time-lapse epifluorescence experiments, (**A**) with exponentially growing RecJ-CFP cells 45 min after induction of DSBs with 100 ng/ml MMC. Pictures were taken every minute. White box indicates area enlarged on the right. White arrowheads indicate localization of static RecJ-CFP foci, grey arrowheads dynamically appearing and disappearing foci. (**B–C**) Stream acquisition (100 ms intervals) showing the assembly and disappearance of RecN-YFP centers. Note that foci arise from several molecules, because experiments are done by epifluorescence, not by SMT! Outlines of cells are indicated by dashed lines. (**B**) Disappearance of single RecN-YFP assembly, (**C**) appearance of a RecN-YFP focus at a different site in the cell following the disappearance of an earlier focus. Scale bars 2 µm.

Induction of DNA damage through MMC results in the formation of visible RecN-YFP foci, when conventional epifluorescence is employed^16^, implying that RecN assembles at distinct sites on the nucleoid as multimers. We aimed at determining the half-life of RecN assemblies and at investigating if they assemble to a single or several sites on the chromosome. From 23 experiments, using 100 ms stream acquisitions under epifluorescence excitation (see suppl. movies 3 and 4 for examples), we found that RecN-YFP foci arose and disintegrated at the frame of few seconds and at different positions within the cell (Fig. 7B,C). Figure 7C shows the disappearance of a RecN-YFP assembly, and the establishment of a new assembly at a different position on the chromosome. On average, RecN-YFP foci persisted for 2.5 ± 0.6 seconds (n = 28 foci analyzed). Similar stream acquisition of different sites on the chromosome tagged with a *lacO*/LacI-CFP array did not show any significant movement of DNA sites 30 to 60 minutes after addition of MMC, using identical acquisition times (suppl. movies 5 to 8). Therefore, RecN assembles at sites of DNA damage for few seconds only, and at many positions on the nucleoid, suggesting that this step of presynapsis occurs throughout the chromosome, similar to RecJ, which moves from being bound to the replication machinery (one or two foci per cell) to forming many foci on the nucleoids (Figs 7A and S3). As shown by time lapse microscopy, RecJ-YFP foci appear and disappear between 1 min intervals (Fig. 7A), in agreement with the determined average dwell time of less than 100 ms (only 3% of RecJ molecules arrest for an average of 0.5 s) (Fig. 4b). Therefore, DNA breaks are prepared for RecA loading at their corresponding site within the nucleoid, rather than break sites being recruited to existing repair centres, and are also initiated away from replication forks.

**Figure 8:**
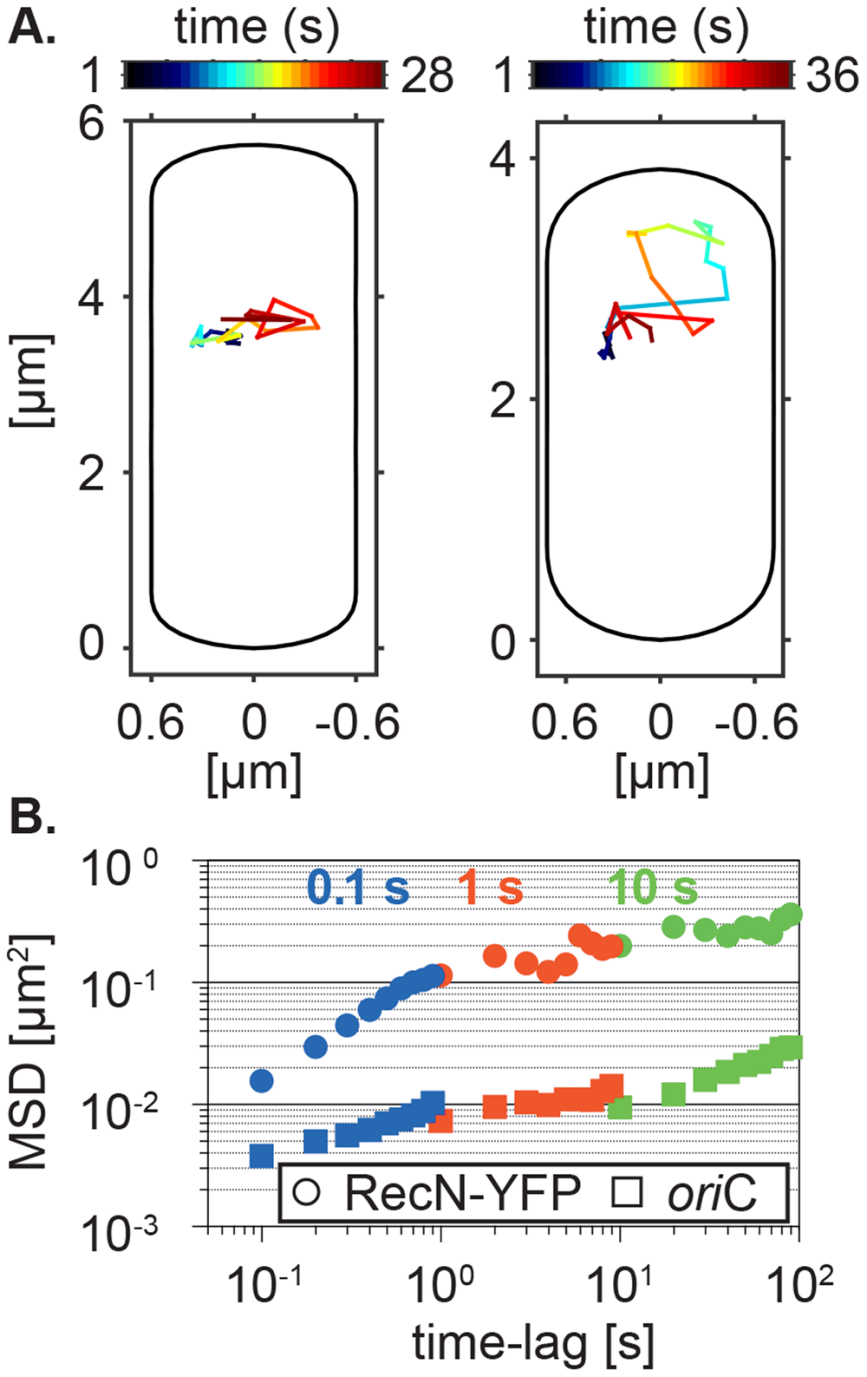
Mobility of RecN assemblies in the presence of DNA damage (**A**). Representative two-dimensional trajectories of RecN-YFP acquired at 1 s time intervals. The color-code corresponds to the time-scale indicated by the color bar. (**B**) Mean-squared displacement analysis of time-lapse microscopy data acquired at different time-intervals for RecN-YFP and the *oriC*-locus following treatment with 50 ng/ml MMC. Shown are time-ensemble-averaged MSDs for the first ten time-lags of the trajectories acquired at each time interval. Only trajectories with a length ≥10 frames were included. To cover the full range of the MSDs curves the data were plotted in a log-log scale.

To quantify the dynamics of RecN assemblies, we performed time-lapse microscopy at varying time-intervals (0.1, 1 and 10 s). Before imaging we induced DNA damage by adding 50 ng/ml of MMC to the growing culture. Figure 8A shows two individual tracks with a lifetime of 28 or 31 s, respectively, acquired at time-intervals of 1 s with short exposure times of 500 ms to minimize bleaching effects of the sample. The assembly associates at the center of the short axis and then moves along the axis to right side, where it then reverses its direction. After the reversal, it moves to the other side of the cell from where it finally diffuses back to its original position (Fig. 8A, left panel). Figure 8A, right panel, shows a trajectory that first rests at its original place of association and then explores a larger space to later come back to its original point of association. To quantitatively describe these time-lapse experiments, we calculated the mean-squared displacements for the first 10 time lags (time-averaged) for trajectories with a minimal length of 10 frames and plotted the MSDs values as an ensemble average (Fig. 8B). We calculated the apparent diffusion coefficients D* = MSD/(2*d*Δt) with dimension d = 2 and Δt = 1 for each time-interval imaged. As expected from the MSD plot, D* linearly decreased with increasing time-intervals and ranged from 0.05 (±0.007 µm^2^ s^−1^ sd) to 0.007 (±0.002 µm^2^ s^−1^ sd), corresponding to very slow movement compared with free diffusion.

Previously, it was shown that assemblies of RecN-YFP overlap with the nucleoid. To exclude that the movement of RecN-YFP after induction of DNA damage originates from the movement of the chromosome, we made use of a fluorescent repressor operator system (FROS). Similarly to the time-lapse microscopy of RecN-YFP, we tracked the locus encoding *spo0J* located in close proximity to the origin of replication (*oriC)* 60 min after induction of DNA damage. In contrast to the motion of RecN-YFP, which shows faster movement and explores a larger area, the mobility of the genomic loci was more confined and slower over the full range of frame rates measured {0.009 (±0.002 µm^2^ s^−1^ sd) − 0.0003 (±3 × 10^−5^ µm^2^ s^−1^ sd)} (Fig. 8B). Taken together, our experiment concerning the mobility of the *oriC* locus first proves that our imaging regime is robust against external perturbations and confirms that the dynamics seen for the assemblies of RecN-YFP do not underlie the motion caused by the chromosome, and that RecN assemblies can move over considerable areas on the nucleoid.

### Transitions between RecN fractions during DSB repair

**Figure 9:**
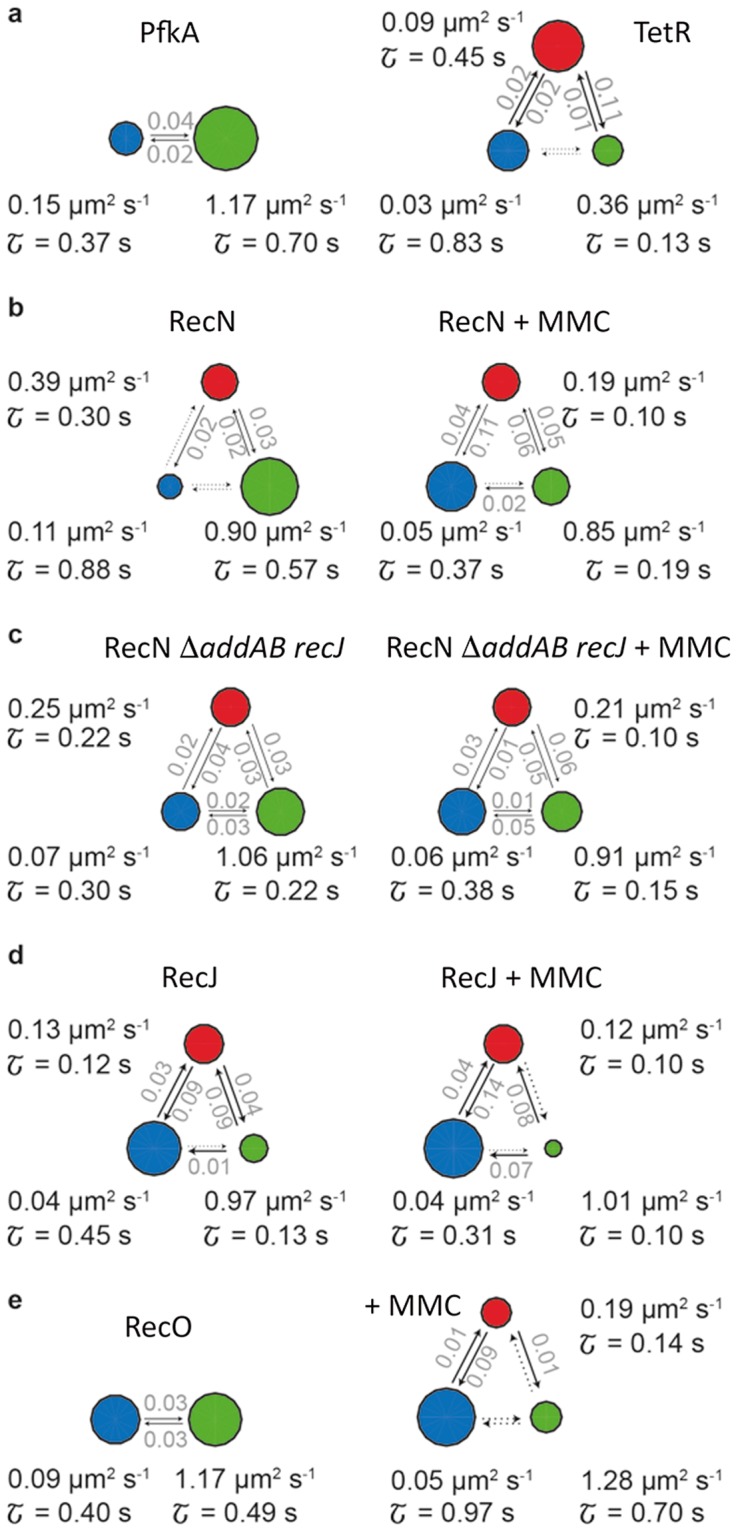
Results of variational bayes single particle tracking (vbSPT) analysis. (**a**) Left: PfkA-YFP; Right: TetR-YFP. (**b**) RecN-YFP in the absence (left) and presence of DNA damage (right). (**c**) Strains deleted for *addAB* and *recJ* expressing RecN-YFP in the absence (left) and presence of DNA damage (right). (**d**) RecJ-YFP in the absence (left) and presence of DNA damage (right). (**e**). RecO-YFP in the absence (left) and presence of DNA damage (right). The vbSPT analysis provides information about the number of diffusive states, the diffusion coefficients, the mean dwell time of each state and the transition probabilities between the states. The circles represent the individual states and the area of the circle indicates its relative occupancy. Arrows and associated numbers in grey show the probabilities that a molecule transitions from one state to the other in one time step. Dotted arrows indicate transitions with very low probabilities.

A hidden Markov model (HMM) algorithm is a powerful tool to model the probability distribution of time series by assuming that an observation at time t is generated by an internal state that is hidden to the observer and that is only dependent on its immediate precursor. We applied HMM integrated in the vbSPT software, devised by the Elf laboratory^25^, to our data (Fig. 9). The vbSPT software uses this model to extract the number of diffusive states, the relative state occupancy, the mean lifetime and the transition rates from the experimental data without prior assumptions. For most FP fusions, the fit revealed three diffusive states except for PfkA and RecO-YFP measured in normally growing cells (Fig. 9a,e). Besides a slow and fast diffusive state that we reported before using the GMM method, vbSPT analysis further suggested an intermediate state for TetR, RecN and RecJ, that ranged from 0.09 to 0.39 µm^2^ s^−1^ in samples not treated with MMC, or from 0.12 to 0.21 µm^2^ s^−1^ in cells treated with a DNA damaging agent (Fig. 9). These data suggest that the DNA repair proteins investigated here are either DNA-bound, scan the DNA through a non-specific DNA scanning mode (intermediate population) or are freely diffusive. Even though the diffusion coefficient of the intermediate state slightly decreased for the repair proteins upon induction of DNA damage, the relative occupancy of the state remained nearly at the same level. This observation is highly interesting, as it suggests that a relatively constant number of e.g. RecN molecules continues to scan the DNA for DNA lesions, while DNA-bound RecN molecules increase at the expense of freely diffusing molecules. Exchange of populations occurs in the way that DNA-scanning molecules find substrate (DNA breaks/repair centres), while freely diffusive molecules convert to a DNA-scanning mode. How the latter conversion is achieved is presently unknown, but clearly, DNA damage alters non-specific DNA binding properties of RecN, ensuring that most molecules present in the cell are optimally employed for the search and preparation of DSBs. Similar to our previous observations, the occupancy of the states with diffusion coefficients representing the slow and the fast fractions of the repair proteins severely changed upon treatment of the cells with MMC. Entry of RecN into the nucleoid (possible through a change in overall DNA structure) can also be seen from the heat map shown in Fig. 4d.

To identify correlations of all data we performed a cluster analysis using the diffusion coefficient and the dwell time (Suppl. Fig. S4). The first cluster represents the slowest moving populations with the longest lifetime (D1 RecN-YFP, RecO-YFP + MMC, TetR-YFP), the second cluster represents all remaining slowly diffusing populations and D2 of TetR-YFP, the third cluster includes all intermediate populations (“D2”) and D3 of TetR-YFP, and the last cluster encloses all fast moving populations represented by the control protein PfkA-YFP. This analysis supports the existence of three distinct populations with different diffusion rates, as suggested by vbSPT.

## Conclusions

In summary, we report on the dynamics of key proteins mediating early steps during HR in real time and at the single-molecule level using SMT, which resolves and quantifies the diffusive states of RecN, RecO and RecJ DNA repair proteins including their diffusion coefficient, their relative contribution to each state, their distribution in the cell and their residence times. While RecJ is present at replication forks^26^, RecN and RecO scan the entire genome, mostly at its periphery, for the presence of DSBs. In the presence of DNA damage, RecN and RecO similarly redistribute from the diffusive/scanning mode to a confined diffusive state, and deeply enter the nucleoids. Even when bound to sites of DNA damage, dwell times of RecN and RecO are relatively short compared to TetR – arguing against a scaffolding function of RecN or RecO, but rather for enzyme-like functions. This agrees with the rather short life time of RecN assemblies of less than 3 seconds. RecN assemblies arise at different sites on the nucleoids, showing that DNA repair centres are set up at many subcellular sites in a time frame of few seconds. For *E*. *coli* RecN, it has recently been shown that it stimulates strand invasion activity of RecA^27^, which may also confer to *B*. *subtilis* RecN based on its highly dynamic behavior, while a genome-wide maintenance of sister chromosome interactions^28^ appears an unlikely function for BsRecN.

We speculate that changes in DNA supercoiling, derived from the formation of DSBs (the nucleoid strongly decondenses after addition of e.g. MMC) mediate the change in RecN diffusion, opening up the DNA to allow more access to RecN. This way, a similar population of molecules continues scanning the chromosome for break sites, while more RecN is engaged in static DNA binding at the expense of freely diffusing molecules. Our data also suggest that RecN is displaced from its binding sites by RecJ or AddAB, so RecN appears to perform a protective function for free 3′ - OH sites to which it binds *in vitro*^18^, possibly avoiding DNA degradation by non-specific exonucleases. RecJ, on the other hand, appears to be freed from replication forks (where it is bound by SsbA^26^) and arrests at many sites on the nucleoids to resect broken DNA ends (Fig. 1). Therefore, presynaptic functions occur throughout the nucleoid, and occur at time scales of seconds or less.

## Material and Methods

### Bacterial strains and media

For fluorescence microscopy, *Bacillus* strains (Supplementary Table S1) were always grown in freshly prepared S_750_ medium^29^ containing fructose as carbon source while keeping the selection pressure using the appropriate antibiotics. To reduce the number of origins of replication, strain CK188 was grown without casamino acids. Strains were first streaked on selective LB plates, inoculated for overnight growth, diluted 1:100 in fresh media and grown to exponential phase at 30 °C. For induction of DSB, cells were treated with 50 ng/ml of MMC for 60 min if not otherwise indicated as in supplementary Fig. S2.

To construct the fluorescent protein fusion RecJ-YFP, 500 bp of the 3′ end of *recJ* were amplified using primer pair RecJ_ApaI_up/RecJ_ClaI_dw and chromosomal DNA of *B*. *subtilis* PY79 as template. The PCR product was digested with *Apa*I and *Cla*I and cloned into correspondingly digested pSG1164y^16^. To generate PfkA-YFP, 500 bp of the 3′ end of *pfkA* were amplified with primer pair pfkA_up/pfkA_dw using chromosomal DNA of *B*. *subtilis* PY79 as template. After digestion of the PCR product with *Apa*I and *Xho*I, *pfkA* was ligated with similarly digested pSG1164y. Plasmids were transformed into competent cells of *B*. *subtilis* PY79 and integrations (occurring at the original gene locus, such that the fusion is driven by the native promoter) were selected on the basis of the corresponding antibiotic.

### Single molecule tracking and image processing

Cells were spotted on coverslips (25 mm, Menzel) and covered using 1% agarose pads prepared before with fresh S_750_ minimal medium by sandwiching the agarose between two smaller coverslips (12 mm Marienfeld). All coverslips were cleaned before use by sonication in Hellmanex II solution (1% v/v) for 15 min followed by rinsing in distilled water and a second round of sonication in double distilled water. Imaging was done with a Nikon Ti-E microscope configured with a motorized stage, a high numerical aperture objective (CFI Apochromat TIRF 100XC Oil, NA 1.49), an EM-CCD camera (ImagEM X2, Hamamatsu) and an appropriate filter set for imaging YFP molecules (YFP HC Filterset; BrightLine 500/24, Beamsplitter 520 and BrightLine 542/27). Specimens were continuously illuminated with a Gaussian shaped laser (TOPTICA Beam Smart, 515 nm, max. power <200 mW) with an intensity of 160–320 W/cm2 and streams were recorded at a frame rate of ~67 Hz in VisiView (Visitron Systems).

Image processing of movies was done in MATLAB (MathWorks) and in the standalone version of Oufti^30^. For each movie, we performed manual segmentation of cells using simultaneously acquired brightfield images in Oufti and then calculated the mean internal fluorescence signal of the cells over time to draw a photobleaching curve. By fitting the curve with a two exponential decay function we determined the time when the sample has reached the single molecule level and thus marking the starting point of the SMT analysis^31^. SMT was performed in U-track^20^. The paramaters used for localization and tracking are listed in Supplementary Table S2. All further analysis was performed with the software SMTracker^32^ and the vbSPT algorithm^25^.

### Gaussian Mixture Modeling to assess diffusive behavior of subpopulations

We determined the mobility of subpopulations and the size of subpopulations based on the frame-to-frame displacements of the molecules in one dimension by fitting a superposition of two Gaussian distributions to the displacement distributions, as described previously^32^. Briefly, for each protein it is assumed that it occurs in two states, e.g., in a free and a DNA-bound state. To fit the experimental data, we thus used a Gaussian Mixture Model with a superposition of two normal distributions with zero means and variances σ_1_ and σ_2,_ respectively, to describe the overall shape of the displacement distributions *P*,

$$P=\alpha \cdot N(0,{\sigma }_{1})+(1-\alpha )\cdot N(0,{\sigma }_{2})$$

where α = f_1_ is a number between 0 and 1, denoting the relative area fraction of the first Gaussian distribution, and f_2_ = 1 − α is the relative area fraction of the second Gaussian distribution. The variances σ_1_ and σ_2_ are directly related to the diffusion coefficient by $D=\frac{{\sigma }^{2}}{2\times {\rm{d}}\times {\rm{\Delta }}t}$, with *d* representing the dimension and *Δt* corresponding to the time interval used for tracking. To compare the diffusive behavior of proteins in normally growing cells and cells treated with MMC, we perform simultaneous fits to the displacement distributions, where the only parameters that change are the fraction sizes f_1_ and f_2_. To fit this model to our data, we minimized the residual sum of squares (*RSS*) between the empirical cumulative distribution functions (ECDF) of the experimental data and the Gaussian Mixture Model, by using a trust-region reflective Newton method implemented in our software.

### Calculation of residence times of molecules and classification in confined or free movement

To determine the residence time of the molecules, we count the time a molecule stays inside a radius of defined size. The radius is defined before by the Gaussian Mixture Model. Here, the standard deviation of the normal distribution corresponding to the smaller displacements represents the immobile population. We set 99% of the smaller displacements (≅3*σ_D1_) as an upper threshold to distinguish slow from fast movement. Therefore, we first calculate the Euclidean distance between consecutive localizations *n* for all displacements in the trajectory and then compute the time *t* that the molecule stays in the slow movement state such that *t* = (n − 1)*t*_exposure_. The inverse empirical density function of *t* is then plotted and the distribution is fitted using a one- and two-component exponential decay function:

$$f(t)=a\ast {e}^{-\lambda \ast t}$$

and

$$f(t)=a\ast {e}^{-{\lambda }_{1}\ast t}+(a-1)\ast {e}^{-{\lambda }_{2}\ast t}$$

to obtain the decay constants λ (Eq. S1), λ _1_ and λ _2_ and its contributions (*a* and *a − 1*) (Eq. S2). The final residence time is then calculated as $\tau =\frac{1}{\lambda }$.

Based on the computation of the time *t* that a molecule stays in a slow state, we also classified the trajectories into immobile and mobile trajectories. If all consecutive displacements of a trajectory were located inside the radius around the original site of appearance, the molecule was classified as immobile and plotted in red into a standardized cell. Only tracks with at least 4 consecutive localizations were included in the analysis. To create 1D histograms of immobile and mobile tracks along the short and long axis of the cell, the localizations of the classified tracks were binned and plotted as probability density functions.

## Electronic supplementary material

Supplementary Dataset 1
