# Supplementary material for: Single molecule tracking reveals spatio-temporal dynamics of bacterial DNA repair centres
Source: Sci Rep. 2018 Nov 6;8:16450. doi: 10.1038/s41598-018-34572-8 (PMC6219548; doi:10.1038/s41598-018-34572-8)
Supplement: Supplementary file 1 — Supplementary Dataset 1 [file 41598_2018_34572_MOESM1_ESM.pdf]

Single molecule tracking reveals spatio-temporal dynamics of bacterial DNA repair centres

Thomas C. Rösch[1, 2], Stephan Altenburger[1, 2], Luis Oviedo-Bocanegra[1, 2], Miriam Pediaditakis[1, 2],
Nina El Najjar[1, 2], Georg Fritz[1,3] and Peter L. Graumann[1, 2*]

[1]SYNMIKRO, LOEWE Center for Synthetic Microbiology, Marburg, Germany; [2]Department of Chemistry, Philipps Universität Marburg, Germany; [3]Department of Physics, Philipps Universität Marburg, Germany

Supplementary Material

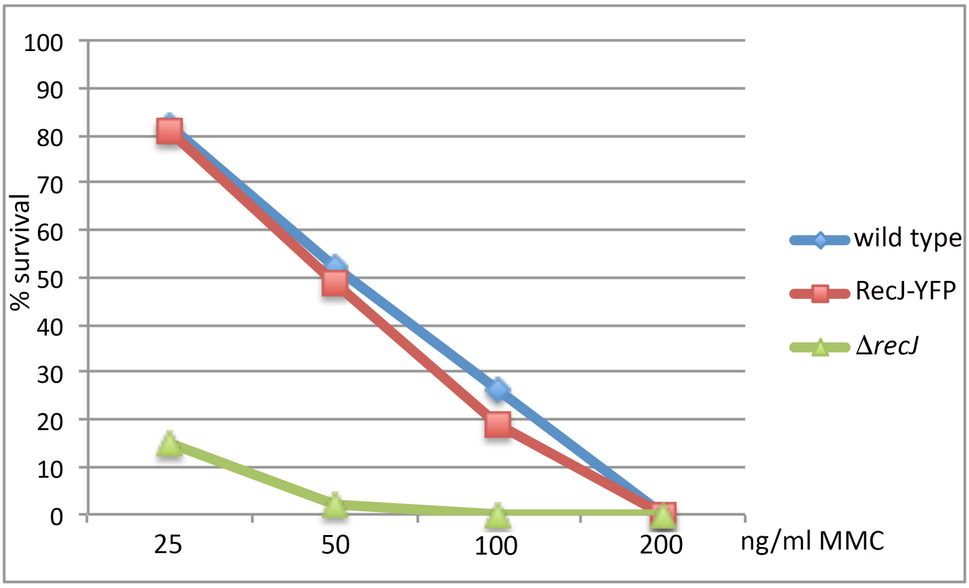

Figure S1 Survival (in %) in response to 60 minute-treatment with different concentrations of Mitomycin C. Δ*recJ*: Cells carrying a deletion of the recJ gene.

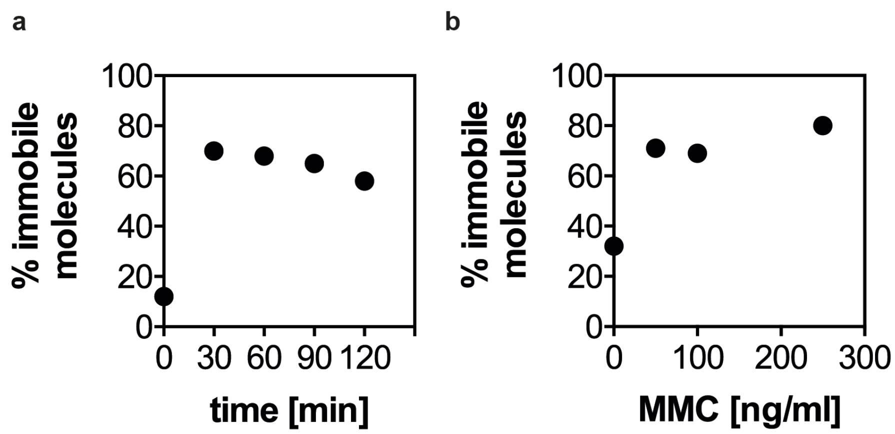

Figure S2 Time-course and dose dependent SMT of RecN-YFP after induction of DNA damage. (**a**) Time lapsed SMT of RecN-YFP before and after induction of DNA damage using 50 ng/ml MMC. (**b**) SMT of RecN-YFP at varying concentrations of MMC (0, 50, 100 and 250 ng/ml) after continuing growth for 30 min.

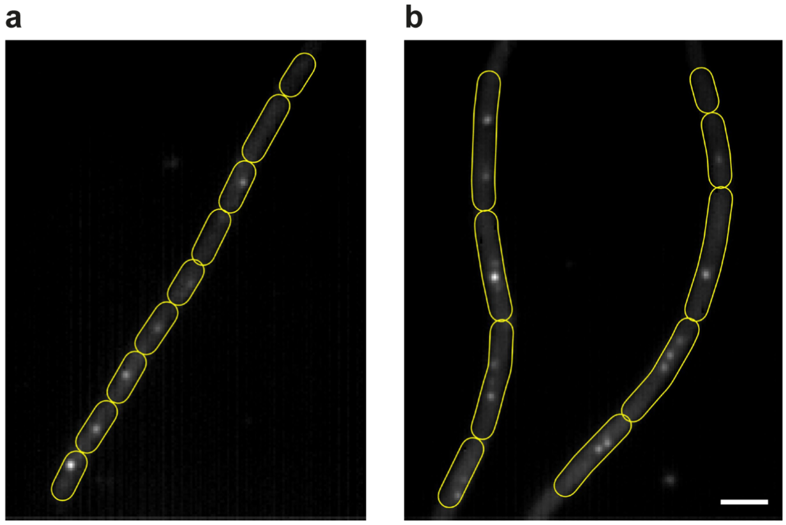

Figure S3 Redistribution of RecJ-YFP in cells treated with MMC. RecJ-YFP before (**a**) and after induction of DNA damage (**b**) using 50 ng/ml MMC. Images are overlays of all frames of stream acquisitions, showing the position of static fractions. In exponentially growing cells. RecJ-YFP is bound at the usually centrally positioned replication machinery. After induction of DSBs, frequently more than 2 static assemblies are present, although no more than 2 replication forks are present in the cell. White bar 2 µm.

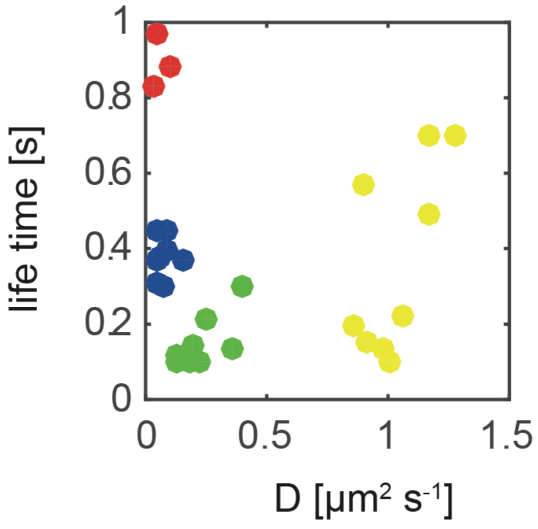

Figure S4 | Cluster analysis of vbSPT data. Scatterplot of all proteins under study showing the relation of lifetime of the state and the diffusion coefficient of the state. To find clusters we used a Gaussian mixture model analysis and the Bayes Information Criterion (BIC) to determine the best model fit. The cluster analysis reveals 4 clusters: Highlighted in red are those with a low D, but long lifetimes (D1 of RecN-YFP, RecO-YFP+MMC and TetR-YFP). In blue are shown samples with low D and intermediate lifetimes (D1 of RecN-YFP+MMC, RecN-YFP±MMC in *addAB recJ* mutant, RecJ-YFP±MMC, RecO-YFP, PfkA-YFP and D2 of TetR-YFP). The green cluster represents those samples with intermediate D and lifetime (D2 of RecN-YFP±MMC, RecN-YFP±MMC in *addAB recJ* mutant, RecJ-YFP±MMC, RecO-YFP+MMC and D3 of TetR-YFP). The yellow group finally encloses all remaining fast states (D3).

Supplementary Table S1 | Strains used in this study

Strain	Genotype (resistance)[a]	Reference
B. subtilis		
PG600	*recN-yfp tet*	{Kidane:2004fy}
PG665	*recN-yfp addAB recJ cm tet*	{Sanchez:2005dw}
PG760	*recO-yfp tet*	{Kidane:2004fy}
PG1387	*recJ-yfp cm*	This work
PG2664	*pfkA-yfp cm*	This work
CK83	*spo0J::tetO array* (*kan*^{R}) *cgeD::*P_{pen} *tetR-yfp* (*tet*^{R})	{Kaimer:2009kx}

[a] *cm*, chloramphenicol (5 µg/ml); *tet*, tetracycline (5 µg/ml); *kan*, kanamycin.

Supplementary table S2: Statistics of SMT experiments

	**RecN-YFP**		**RecN-YFP *addAB recJ***		**RecJ-YFP**		**RecO-YFP**		**PfkA-YFP**	**TetR-YFP**
MMC	-	+	-	+	-	+	-	+	-	-
# movies	10	11	10	6	6	7	12	9	7	5
# cells	48	54	72	20	65	44	50	26	37	15
length* [µm]	3	3.62	2.35	3.6	2.19	4.74	4.79	6.16	3.1	4.24
# tracks	519	676	656	287	700	962	195	226	237	1755
lifetime** [s]	0.142	0.183	0.103	0.153	0.153	0.195	0.0975	0.14	0.098	0.302

* and **: Shown is the mean cell length or the mean lifetime of the tracks.

Supplementary table S3: Results of the vbSPT analysis

	RecN-YFP		RecN-YFP *addAB recJ*		RecJ-YFP		RecO-YFP		PfkA-YFP	TetR-YFP
MMC	-	+	-	+	-	+	-	+	-	-
D1	0.11 ± 0.00	0.05 ± 0.00	0.07 ± 0.00	0.06 ± 0.00	0.04 ± 0.00	0.04 ± 0.00	0.09 ± 0.00	0.05 ± 0.00	0.15 ± 0.01	0.03 ± 0.00
D2	0.39 ± 0.01	0.19 ± 0.00	0.25 ± 0.00	0.21 ± 0.00	0.13 ± 0.00	0.12 ± 0.00	-	0.19 ± 0.01	-	0.09 ± 0.01
D3	0.90 ± 0.02	0.85 ± 0.02	1.06 ± 0.03	0.91 ± 0.03	0.97 ± 0.03	1.01 ± 0.04	1.17 ± 0.05	1.28 ± 0.07	1.17 ± 0.04	0.36 ± 0.01
A1	11.7	47.1	27.5	42.2	55.8	66.1	46.1	64.2	21.2	31.8
A2	25.3	26.7	28.7	27.1	29.3	28.5	-	17.3	-	50.7
A3	63.0	26.2	43.8	30.7	14.9	5.4	53.9	18.5	78.8	17.5
τ_1 [s]	0.882	0.372	0.299	0.376	0.451	0.311	0.396	0.972	0.366	0.830
τ_2 [s]	0.298	0.097	0.217	0.098	0.117	0.099	-	0.144	-	0.451
τ_3 [s]	0.573	0.192	0.220	0.154	0.132	0.098	0.490	0.703	0.697	0.135
P_{12} [s-1]	0.006*	0.036	0.024	0.028	0.031	0.043	0.035	0.011	0.036	0.018
P_{21} [s-1]	0.020	0.105	0.04	0.099	0.090	0.142	0.027	0.093	0.019	0.019
P_{13} [s-1]	0.008*	0.004*	0.025	0.011	0.002*	0.005*	-	0.003*	-	0.000*
P_{31} [s-1]	0.001*	0.016	0.033	0.044	0.014	0.074	-	0.007*	-	0.001*
P_{23} [s-1]	0.029	0.050	0.029	0.056	0.039	0.010	-	0.012	-	0.014
P_{32} [s-1]	0.025	0.063	0.035	0.053	0.099	0.081	-	0.009*	-	0.110

* rare transitions have transition probabilities < 0.001
